# The surface of the eye – a superficial entity with deep repercussions

**Published:** 2009

**Authors:** Vasile Potop, Marieta Dumitrache, Alina Ciocalteu

**Affiliations:** * The Clinical Ophthalmologic Emergency Hospital, Bucharest

**Keywords:** surface of the eye, autologous serum, amniotic membrane, keratoconjunctivitis sicca

## Abstract

The surface of the eye is an anatomical and functional entity with a relatively recent delimitation but with significant therapeutic and diagnostic consequences. The pathology of the conjunctive and cornea must be approached by looking at the interrelations between the two tissues that are so different anatomically and functionally but in the same time form a unit in structuring the eye’s surface. There are two major categories of relations between the two tissues: one of them is mediated by lachrymal secretion, a process whose complexity is not yet fully understood, and the other is germinal, referring to the stem cells located at the limbus which become epithelial cornea cells that can fixate lachrymal fluid.

Imbalances in the quantity and quality of lachrymal secretion can be compensated, up to a certain point, by artificial products, but in severe cases only specially prepared autologous serum can compensate the deficit.

The limbic deficits that affect stem cells require complex therapeutic procedures like limbic cell transplant, using an amniotic membrane or autologous serum.

The ocular surface, a relatively recent entity in the field of ophthalmology, highlights the functional unit of the cornea and the conjunctive.

The two are anatomically different tissues but they form a unit of seemingly contrary elements, with complex functional implications, that expresses the physical, chemical and biological integrity of the eye in the exterior environment that permanently withstands mechanical, chemical and bacterial aggression.

Although the aggressions they are subjected to are the same, the cornea and the conjunctive have different defensive “behaviors”.

The cornea is an avascular tissue with a regulated internal structure that ensures its transparency. It doesn’t have its own means to defend itself and any inflammatory conflict can have repercussions on its transparence and geometry. Its defensive capacity is ensured indirectly by the conjunctive through tears.

The conjunctive is a profusely supplied tissue, with blood vessels that are connected to lymph vessels rich in immune cells. It has good immune reactivity and its transparence and geometry are not functionally important. Besides its defense possibilities, the conjunctive also protects the eye’s surface by producing tears and controlling the output both qualitatively ant quantitatively.

The lachrymal film is the uniform dispersion of tears on the surface of the cornea, having a total thickness of about 40 microns. The lachrymal film is classically described as having three layers (an inner mucous layer, an intermediate watery layer and an external fatty layer).

The first two ensure the adherence of the watery component in the film to the hydrophobic membranes of epithelial cells in the cornea. In the deeper portion, the concentration of mucus is high and gives a mucous jelly-like consistence to the structure also providing some cohesion, which protects against the forces produced by the blinking motion. The lachrymal film ruptures, leaving a 30-micron later intact and a 10-micron watery layer with low mucus concentration. This progressive reduction of mucus concentration from the deep to the superficial portions of the lachrymal film determines where the film ruptures during blinking and softens the ripping forces of the eyelids on the epithelium in the cornea.

The fatty layer is produced by Meibomius glands and is approximately 100nm thick. It prevents evaporation between blinks, reduces the superficial tension of the tears and ensures the optimization of the cornea’s optical qualities.

The lachrymal film defends the surface of the eye through immune defense factors ( secretor A immunoglobulin produced by plasmocytes in the conjunctive and lachrymal glands, factors of the serum complement), mechanical defense factors (washing away micro-organisms and foreign bodies) and chemical defense factors (lactoferrin and lysozyme). 

Although the inflammatory reaction is, to a certain degree, a defensive reaction, the lachrymal film contains anti-inflammatory and anti-proliferative factors that play a role in the regeneration and differentiation of different cell types. A series of immune modulation factors TGFbeta1, TGFbeta2 and trophic factors that play a role in the scarring process (epithelial growth factors, endothelins) ensure the parameters of the immune response and scarring process of the cornea, so as its anatomical integrity and transparency are priority.

Substitutes for natural tears can ensure the deficit of water flow and model evaporation and drainage but they cannot ensure the immune and modulation components or the growth factors, which are secreted in a biological context; they have a short lifespan and local action. For all these functions the only solution is autologous serum.

The deficit of lachrymal film, besides the various disorders caused by the loss of its functions, determines the synthesis of pro-inflammatory cytokines that, in turn, can affect calceiform cells, developing induction circles that further worsen the functional deficit.

The lachrymal and Meibomius glands are receptors for different steroid hormones, androgens, estrogens and progesterone but also prolactin, TSH, LH and FSH in the conjunctive. The eye is a target-organ for these hormones that have trophic functions and play a part in the homeostasis of the lachrymal film.

Minor androgens have a major role in the normal function of the Meibomius glands but they are also immune modulators and immunosuppressant. 

The cornea contains about 60 times as many nerve endings as the dental pulp. Main lachrymal glands, but also accessory and Meibomius glands have rich innervations, especially from the parasympathetic component in which the mediators are acetylcholine and the vasoactive intestinal peptide (VIP), but also sympathetic innervations mediated by noradrenalin and a sensitive one mediated by the P substance and **calcitonin gene related peptide (CGRP)**. Acetylcholine determines a rise in the secretion of proteins and water but also of lactoferrin and epithelial growth factor. The release of VIP determines increased mucus secretion.

At tissue level, the interrelations between various neuromediators are complex, their actions follow one another in a cascade and the affection of any one of the steps can lead to a global decrease in lachrymal secretion. This is why the inflammatory infiltrate in some autoimmune diseases can alter local innervations and consequently affect tear secretion, diminishing it.

Chronic inflammatory reactions can appear as a result of hormone disorders or starting from simple corneal dryness.

Dehydration of the ocular surface triggers nervous stimulation to regulate lachrymal secretion and repair lesions. Neurogenic stimuli determine the release of cytokines at the level of the conjunctive, lachrymal gland and lachrymal film. These in turn attract cells that are involved in the cellular immune response that will cause a chronic cytotoxic inflammatory reaction. Thus, a simple desiccation through excessive evaporation turns into a chronic disease that destroys lachrymal glands and calceiform cells. 

As the inflammatory infiltration grows, nervous control of the main lachrymal gland decreases and the situation worsens progressively.

In cases where minor androgens are insufficient (in menopause), because of their role as immunosuppressant, chronic inflammation can appear, as well as cell apoptosis in the lachrymal glands.

Regardless of the initial disorder, be it autoimmune or hormonal, it can decompensate either iatrogenically through aggressive treatments, external factors (pollution), local infections or allergic phenomena and the eye’s surface suffers a series of cellular or hormonal reactions that break down its protection systems, disrupt the lachrymal film and prolongs the suffering of epithelial cells in the cornea and conjunctive by means of a vicious circle that closes, making it impossible to detect the initial link.

Sjorgren syndrome is an autoimmune reaction that destroys the main lachrymal gland through fibrosis and diffusely infiltrates the conjunctive with inflammatory factors.

Antinuclear antibodies can block M3 muscarinic receptors, inhibiting lachrymal secretion through a local parasympathetic block. 

Some types of chronic allergic conjunctivitis can trigger the activation of the local immune system with cytotoxic effects on the conjunctive’s epithelium and repercussions on the lachrymal film, affecting its stability by destroying calceiform cells.

There are certain forms of conjunctive allergies with an apparently diminished inflammatory reaction but with intense symptoms and a significant change in the “ripping time” of the lachrymal film.

There is an association between the symptoms and phases of allergy. (pruritus is the main symptom)

Often after an acute conjunctivitis or a viral keratoconjunctivitis there is cloudiness in the lachrymal film that often concerns the patient more than he initial disease. This is a consequence of the fact that calceiform cells were affected during the inflammatory reactions triggered either by the disease or by its treatment. 

The same situations can be found in chronic infections. A particular example is Chlamydia where there are no clinical signals on one side, while the other side exhibits severe ocular dryness.

Blepharitis and rosacea are accompanied by qualitative keratoconjunctivitis sicca with infectious and inflammatory determinism. These affections determine dysfunctions in the Meibomius glands and affect the fatty layer of the lachrymal film and cause rapid evaporation of tears. Eventually, there is a shortening of the time it takes for the film to brake, epithelial hiperosmolarity and ultimately chronic inflammation on the eye’s surface.

The same dysfunction of he Meibomius glands can lower the fluency of their secretion and cause retention of the content and infection with white saprophyte staphylococcus, which is not pathogenic in normal conditions but in such situations it partially metabolizes the lipids, which leads to the accumulation of fatty acids which are irritating and pro-inflammatory.

Each administration of eyewash causes a slight corneal-conjunctive discomfort. Sometimes, this discomfort is more severe and can be seen as a superficial dotted keratitis especially in the nasal area, where the eyewash accumulates. 

Eyewashes affect the surface of the eye because of the various preservatives in their composition, which have a cytotoxic effect on the ocular surface. Such preservatives like those based on quaternary ammonium bases also act as detergents, altering the fatty component of the lachrymal film and accelerating its evaporation. They also affect the ability of microvillus in the epithelium of the cornea to adhere to tears. Benzalkonium chloride is known for its cytotoxic effects. In cell culture studies, it was demonstrated that concentrations far smaller than those used in eyewashes induce apoptosis and in regular quantities they even induce cell necrosis. 

At concentrations as small as the hundredth time of those in eyewashes, the cytotoxic effects persist. The half-life of the conservative after administration is about 20 hours and after 160 hours, the concentration is still high enough to provoke toxicity.

The treatment method of replacing eyewash with different types of artificial tears can work up to a certain point. These will make up for the lack in watery component and for part of the lipidic one thus preventing evaporation.

In severe deficits with persistent epithelial defects that do not respond to treatment by substitution and conservation of natural tears, substances prepared from autologous serum substitute the biological functions of humoral defense or stimulation through growth factors.

*The best biological substitute for natural tears has proved to be, up until now, autologous serum introduced for the first time in 1984 by Fox and Tsubota in dry eye therapy for its epitheliotrophic properties*.

Fibronectin, vitamins and epithelial growth factors were used to sustain scarring phenomena but their stability is what ultimately reduced their applicability. These factors exist in human serum; they are stable and very available biologically, while having reduced antigenicity. 

*The complex relation between the conjunctive and the cornea, mediated by the lachrymal film is fluid from a physical perspective. This completes a solid relation where certain structures in the conjunctive generate and modulate the phenotype of epithelial cells in the cornea following strict tissue dynamics*.

The epithelium in the cornea is constantly renewing itself. At the very base of this process are cell division, cell migration and cell differentiation phenomena.

In 1981, Shapiro, Friend and Thoft came with the hypothesis that the epithelium in the cornea really originates in that of the conjunctive, based on observed regeneration of the cornea following complete abrasion of its epithelium.

In 1983, Thoft and Friend revised the theory and suggested that what really explains the regeneration is a migration of cells from the conjunctive to the cornea. During this migration, conjunctive cells turn into corneal cells. (Cellular transdifferentiation)

This theory of cell transdifferentiation was contested following animal experiments in which the epithelia of both the cornea and the conjunctive were destroyed, but the basal limbic layer was left intact.

Today, we accept the theory of limbic stem cells with a role in the continuous renewal of corneal epithelium. This theory was confirmed by studies using thymidine and immunofluorescence concerning the rate of cell mitosis in the basal layer of the limbic conjunctive. The source of the cells in the corneal epithelium is in the basal layer of the limbic, more precisely in the stem cells, which are undifferentiated cells in a slow cell cycle. 

The division of these cells regenerates their own number on one hand, and on the other, it ensures a supply of transitory amplification cells that migrate centripetally to the place they form the basal epithelial layer of the cornea and where they have an intense mitotic activity and following a series of successive multiplication cycles they gain the phenotypic characteristics of corneal epithelial cells.

These, in turn, migrate vertically towards the surface of the cornea, lowering their multiplication rate and gaining the characteristics of differentiated cells for the surface of the cornea, with a role in the anatomical integrity and fixation of the lachrymal film.

During these cell migrations, there is also a cell differentiation based on a genetic program responsible for the properties of superficial corneal cells whose expressivity is subject to local induction. 

The differentiation of limbic stem cells is possible due to the contact with a basal membrane which contains a type of collagen other than that found in the membrane of limbic conjunctive cells (corneal collagen type IV) and also because of the existence of possible neuromediator or hormone receptors, which would explain clinical observations regarding limbic dysfunctions in endocrine or neurological diseases.

Tseng and his collaborators described limbic deficiency as a symptom in 1995. There are two main types of limbic deficiencies:

*Type I limbic deficiency* appears as a result of the direct destruction of limbic stem cells in the following situations: severe burns caused by heat or chemicals, ocular pemphigoid , Stevens-Jhonson syndrome, Repeated surgery on the limbus (cryotherapy, anti-glaucoma surgery with local administration of antimitotics), prolonged use of contact lenses. Treatment: limbic cell transplant – limbus graft.

*Type II limbic deficiency* develops insidiously and progressively in the following situations: neurotrophic keratitis, chronic keratitis, pterygium or chronic limbus inflammations. Treatment: typically steroid anti-inflammatory medication and in advanced stages, limbic transplant. The latest research has revealed that the stroma beneath the limbic stem cells plays an important part in the onset of this type of deficiency, as it can be affected by local hormonal, inflammatory or neurologic influences. The effect is a change in the series of local inductions and consequently in the transformation of limbic stem cells.

In light of these new data, the perspective of treating limbic deficiencies goes beyond the sole option of limbic transplant. The treatment of limbic insufficiency will have to solve: 

- The absence of limbic stem cells

- Destruction or inflammation of the basal membrane in corneal epithelium and of the corneal stroma

- The lack of epithelial growth factors and humoral corneal defense factors

 A new therapeutic method can solve the affection of the basal membrane in corneal epithelium and of the corneal stroma – the human amniotic membrane.

**The use of human amniotic membrane in repairing the surface of the eye**

The amniotic membrane is a fetal membrane of ectoblastic origin that has: a single-layer cubic epithelium, a basal membrane containing type IV collagen and an avascular connective stroma.

The epithelium is destroyed during processing and is not of interest; only the basal membrane is used. In severe lesions of the cornea involving the epithelium, basal membrane and stroma, limbic transplant procedures alone are ineffective.

Tseng and his collaborators demonstrated the relation between the corneal stroma, limbic stem cells and corneal epithelium by using cytokines. Prolonged exposure of the corneal stroma causes the apoptosis of keratocytes with the release of proteolytic enzymes that determine corneal autolysis. 

The basal membrane of the corneal epithelium has the following functions: it induces cell differentiation through the expression of cytokeratins, it ensures the adherence of epithelial cells and inhibits apoptosis. The amniotic membrane has the advantages of a basal membrane that keeps all these functions and can fully replace a damaged one.

The advantages of the human amniotic membrane: good immune tolerance, no histocompatibility antigens, it inhibits neovascularization, it ensures the differentiation, migration and adherence of epithelial cells, it can be a substrate for stem cells, it inhibits tissue proteases and protects the epithelium from subjacent inflammation ensuring a good tolerance of limbic grafts. 

With all the cutting-edge surgical procedures, repairing the ocular surface after it has been severely affected by chemical burns or invalidating diseases like ocular pemphigoid or Stevens-Jhonson syndrome is still a major therapeutic challenge. Besides limbic transplant and using amniotic membranes there is still one problem to solve: ensuring the connective and integrating element between the conjunctive, the germinal sector (limbus) and the cornea.

This is the purpose of natural tears, a “live” product with multiple functions but absent in these severe situations. Up to now, the closest substitute for natural tears has been autologous serum.

The surface of the eye is a complex biological component, whose function satisfies true geometrical requirements necessary for the optical functions of the cornea, it ensures the integrity of the most sensitive tissue (the cornea) in the outside environment, giving it the privilege of not being perceived, it creates the harmony of two anatomically different but functionally united structures, by giving us the delicate window through which we see the world – the lachrymal film. The ocular surface is also a place where systemic pathologies discretely express themselves, from endocrine affections to autoimmune diseases.
